# Cytokine-induced killer cell treatment is superior to chemotherapy alone in esophageal cancer

**DOI:** 10.3389/pore.2023.1610710

**Published:** 2023-06-05

**Authors:** Jiayang Sun, Yushu Sun, Miniderima, Xiumei Wang

**Affiliations:** ^1^ Department of Thoracic Surgery, Affiliated Hospital of Inner Mongolia Medical University, Hohhot, Inner Mongolia, China; ^2^ Department of Oncology, Inner Mongolia Cancer Hospital and Affiliated People’s Hospital of Inner Mongolia Medical University, Hohhot, Inner Mongolia, China

**Keywords:** esophageal cancer, immunotherapy, cytokine-induced killer cells, dendritic cells, network meta-analysis

## Abstract

**Background:** The therapeutic efficacy of cytokine-induced killer (CIK) cells versus dendritic cells (DC) co-cultured with CIK cells (DC-CIK) in treating esophageal cancer (EC) remains unclear due to the absence of a direct comparison of these two regimens. This study evaluated the comparative efficacy and safety of CIK cells versus DC-CIK using network meta-analysis in treating EC.

**Material and methods:** We identified eligible studies from previous meta-analyses, then conducted an updated search to retrieve additional trials between February 2020 and July 2021. The primary outcomes included overall survival (OS), objective response rate (ORR), and disease control rate (DCR), and the secondary outcomes included quality of life improved rate (QLIR) and adverse events (AEs). A network meta-analysis of 12 studies was conducted using ADDIS software.

**Results:** Twelve studies were identified, including six comparing CIK or DC-CIK plus chemotherapy (CT) with CT alone. Immunotherapy plus CT significantly improved overall survival (OS) (odds ratio [OR] 4.10, 95% confidence interval [CI] 1.23–13.69), objective response rate (ORR) (OR 2.72, 95% CI 1.79–4.11), disease control rate (DCR) (OR 3.45, 95% CI 2.32–5.14), and quality of life improvement rate (QLIR) (OR 3.54, 95% CI 2.31–5.41). DC-CIK+CT decreased the risk of leukopenia compared with CT alone. However, no statistical difference was detected between CIK-CT and DC-CIK+CT.

**Conclusion:** Based on the available evidence, we concluded that CIK cell treatment is superior to CT alone, but CIK-CT and DC-CIK+CT may be comparable in treating EC. However, comparing CIK-CT and DC-CIK+CT is only based on indirect evidence, so it is undoubtedly necessary to conduct studies to compare CIK-CT with DC-CIK+CT in EC patients directly.

## Introduction

Esophageal cancer (EC) is one of the most common digestive malignant tumors. It has been reported that approximately 604,100 new EC cases and 544,076 deaths resulted from EC in 2020 [1]. Incidence and mortality of EC in China remain challenging, which is more than global statistics [2]. Surgery, radiation therapy (RT), and chemotherapy (CT) have been most extensively applied to treat EC patients [3]; however, the application of these therapeutic regimens was greatly limited due to the failure to thoroughly eliminate tumor cells, drug resistance, and other adverse reactions [4, 5]. Therefore, developing newer effective, safer therapeutic strategies for EC is imperative.

Studies suggest that immunodeficiency plays a crucial role in the relapse and metastasis of EC [6]; thus, immunotherapy attracts extensive attention and has been widely investigated worldwide. More importantly, immunotherapy has been regarded as the fourth most powerful treatment strategy following surgery, RT, and CT [5]. Among currently available immunotherapy regimens, adoptive cellular immunotherapy protocols, such as natural killer cells (NK) [7], tumor-infiltrating lymphocytes (TILs) [8], cytotoxic T lymphocytes (CTLs) [9], dendritic cells (DC) [10], and cytokine-induced killer (CIK) cells [11] have been flourishing in at-cancer treatment [12]. Compared with other immune cells, CIK cells can be easily obtained from peripheral and umbilical cord blood mononuclear cells. More importantly, it maintains a higher proliferation capacity *in vitro* and has a stronger antitumor activity and a broader spectrum [13].

CIK cells have been extensively applied in at-cancer treatment owing to the following two reasons, including a) the cytotoxicity of CIK cells could not be affected by immune inhibitors [14], and b) CIK cell-mediated cytotoxicity is independent of the major histocompatibility complex (MHC) [15]. Moreover, as the most potent antigen-presenting cells and the essential element for CIK activation, proliferation, phenotype expression, and cytokine secretion [4, 16], the addition of DCs to CIK cells (DC-CIK) further improved the therapeutic efficacy of CIK cells in treating cancer [17]. Several clinical trials have confirmed the therapeutic values of immunotherapy, including CIK or DC-CIK, in treating EC patients [18–20].

It is noted that immunotherapy based on CIK or DC-CIK has been demonstrated to be superior to CT alone for treating EC patients. However, the comparative therapeutic efficacy and safety of CIK versus DC-CIK remain unclear because the study directly comparing these two regimes is absent, significantly limiting the appropriate selection of therapeutic strategies for treating EC patients. It is exciting that network meta-analysis, as an expansion of conventional pairwise meta-analysis, provides a possible strategy for evaluating the difference between the two interventions that were never directly compared. Therefore, we performed this network meta-analysis to assess whether immunotherapy based on CIK+CT significantly differed from immunotherapy based on DC-CIK+CT in therapeutic efficacy and safety for the treatment of EC.

## Materials and methods

This network meta-analysis was conducted according to the recommendations proposed by the Cochrane Collaboration (CC) [21], and all results were reported according to the preferred reporting items for systematic reviews and meta-analyses (PRISMA) for network meta-analysis (PRISMA-NMA) checklist [22, 23]. This study did not require ethical approval and patients’ informed consent because data analysis was performed based on published studies.

### Search strategy

We designed a two-step search strategy to identify eligible studies. Firstly, we identified previously published meta-analyses from PubMed and China National Infrastructure Knowledgement (CNKI) and retrieved eligible studies. In the second phase, two reviewers independently searched PubMed and CNKI to retrieve additional relevant studies published between February 2020 and July 2021. Subject heading terms and complimentary words were used to construct the search strategy. We summarized the search strategy of PubMed in Supplementary Table S1. Any conflicts about the identification of eligible studies were resolved by consulting a third reviewer.

### Study selection

We selected studies using EndNote software according to the following three steps: a) we first removed repeat records by matching the title, author, and journal of each record, b) we initially excluded ineligible records by screening titles and abstracts of remaining unique records, and c) we excluded ineligible studies by checking eligibility based on full texts. We recorded the number of excluded studies and the reasons for excluding each study.

### Selection criteria

According to the previous meta-analyses, we developed the following inclusion criteria: a) patients were confirmed as EC based on histopathology and cytological diagnostic criteria; b) randomized controlled trials (RCTs) contained at least a CT arm and either a CIK+CT arm or a DC-CIK+CT arm; c) studies reported at least one of the six outcome measures of interest, including overall survival (OS), objective response rate (ORR), disease control rate (DCR), quality of life improvement rate (QLIR), adverse events (AEs), including gastrointestinal adverse reaction (GIAR) or leukopenia. Studies were excluded if they met the following criteria: a) abstract without sufficient data; b) Did not report sufficient data; c) repeat studies with insufficient information and poor quality; and d) other treatments such as radiotherapy, target therapy, and Chinese herbal medicine were incorporated into regimes.

### Outcomes

The primary outcomes included overall survival (OS) and treatment efficacy involving objective response rate (ORR) and disease control rate (DCR). The secondary outcomes included quality of life improved rate and adverse events, including gastrointestinal adverse reactions and leukopenia. OS was described as the time from initiating treatment to death from any cause [24]. ORR and DCR were calculated based on some specific indicators, including complete response (CR), partial response (PR), stable disease (SD), or progressive disease (PD). CR plus PR equals ORR, and ORR plus SD equals CDR [20].

### Data extraction

Two independent reviewers used a standard information extraction table designed by our team based on Microsoft Word to extract the essential information: name of the first author, publication year, details of regimes, including culture conditions, cell dose (once), cycles of CIK treatments, timing relative to CT, sample size, age, outcomes of interest, and details of methodological quality. We invited a third reviewer to assist us in resolving any disagreement.

### Assessment of risk of bias

Studies were reviewed for risk of bias by using the Cochrane Collaboration’s risk of bias tool [25] from the following seven items, including random sequence generation, allocation concealment, blinding of participants and personnel, detection bias blinding of outcome assessor, incomplete data, selective reporting, and other bias. In this network meta-analysis, we regarded it as experiencing a high risk if the sample size assigned in each arm of the individual study was less than 30. We invited a third reviewer to assist us in resolving any disagreement.

### Statistical analysis

We first conducted a pairwise meta-analysis built on the random-effects model using RevMan 5.3 (The Nordic Cochrane Centre, the Cochrane Collaboration, Copenhagen, 2014) to establish the role of immunotherapy plus CT for EC. All outcomes in this network meta-analysis were dichotomous variables, and therefore, we used the odds ratio (OR) with a 95% confidence interval (CI) to calculate the pooled results. Heterogeneity was examined using the Chi-square test [26] and I^2^ statistic [27]. Moreover, we investigated the comparative efficacy and safety of CIK+CT or DC-CIK+CT versus CT alone through subgroup analysis.

Following pairwise meta-analysis, we utilized the aggregate data drug information system (ADDIS) software (Groningen, the Netherlands, www.drugis.org) to conduct a network meta-analysis built on Markov Chain Monte Carlo (MCMC) simulation. We set up the following parameters to calculate network meta-analysis: 4 chains, 20,000 tuning iterations, 50,000 simulation iterations, the thinning interval of 10, 10,000 inference samples, and a variance scaling factor of 2.5 [28]. This network meta-analysis was star-shaped, and no loop was constructed; thus, it’s impossible to perform a test for inconsistency [29, 30]. Pooled results in network meta-analysis were presented in OR with a 95% creditable interval (CrI). We utilized the Brooks Gelman-Rubin statistical method to evaluate the convergence, and a potential proportional reduction factor (PRF) of close to 1 indicates achieving a satisfactory convergence [31, 32]. Finally, we also estimated the surface under the cumulative ranking curve to rank all regimes [33]. We did not examine publication bias and small study effects because the number of eligible studies for individual comparison was not more than 10 [34].

## Results

### Identification and selection of study

**FIGURE 1:**
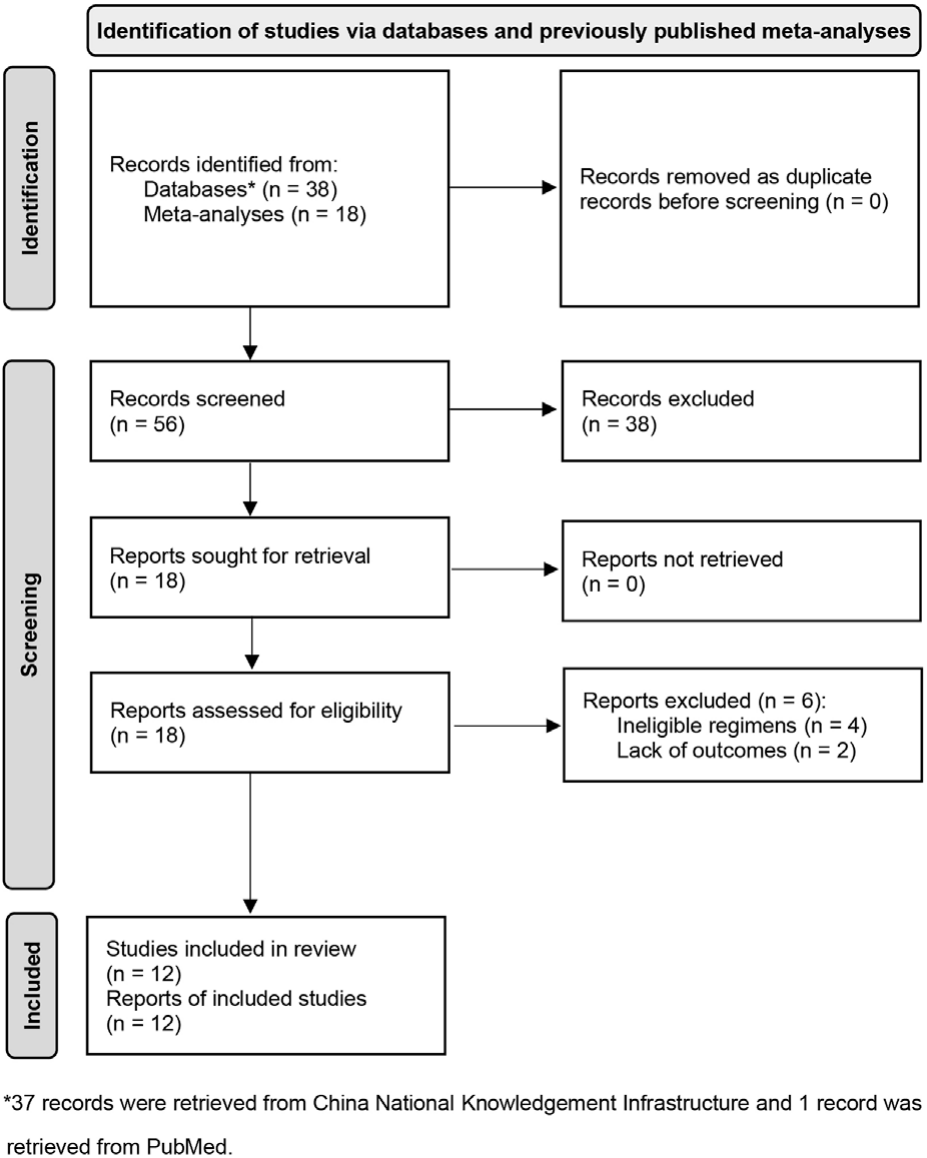
Flow diagram of identification and selection of eligible studies. CNKI, China National Knowledgement Infrastructure; DC, dendritic cells; CIK, cytokine induced killer cells.

We captured three eligible meta-analyses during the initial search phase. Then, a total of 18 potentially eligible studies were identified from them. An updated search in PubMed and CNKI did not identify any additional study. According to our selection criteria, we excluded six for the following reasons: ineligible regimes (*n* = 4) and lack of outcome (*n* = 2). Eventually, 12 eligible studies [35–46] met our inclusion criteria. We used Figure 1 to display the process of identifying and selecting studies.

### Basic characteristics of eligible studies

Among 12 eligible studies included in this network meta-analysis, six studies [35–38, 40, 46] compared CIK+CT with CT, and six studies [39, 41–45] compared DC-CIK+CT with CT. All studies were published between 2010 and 2017 in China. The sample size of individual studies varied from 30 to 100, with an accumulated sample of 1,010. Among these 12 studies, four studies [36, 42, 45, 46] did not report details of cell dose, three studies [36, 41, 42] did not report tumor stage of patients, and four studies [38, 40–42] did not report KPS. Details of all included studies are shown in Table 1.

**TABLE 1:** Characteristics of 12 eligible studies.

Study	KPS	Tumor stage	Sample size	Age (mean or median)	Exp regime	Timing relative to CT	Culture conditions	Cell dose (once), cycles	Infusion model	Source	Outcomes
Chang 2013	>70	III–IV	33 vs. 33	66.0 vs. 66.0^▲^	CIK+CT	2 days	IFN-γ, IL-2, OKT-3	1.0 × 10^9^, 2 cycles	ivgtt.	APB	ORR, DCR, QLIR, GIAR, LP
Gu 2013	>60	n.r.	15 vs. 15	62.0 vs. 64.0^▲^	CIK+CT	7 days	IFN-γ, IL-2, OKT-3	n.r.	ivgtt.	APB	ORR, DCR, QLIR
Liu 2011	>70	III–IV	20 vs. 20	62.0 vs. 62.0^▲^	CIK+CT	2 days	IFN-γ, IL-2, OKT-3	>(1.0–2.0) × 10^9^, 3 cycles	ivgtt.	APB	OS, ORR, DCR, QLIR, GIAR, LP
Qu 2015	n.r.	IV	100 vs. 100	56.3 vs. 56.3^▼^	CIK+CT	14 days	IFN-γ, OKT-3	5.0 × 10^9^, 4 cycels	ivgtt.	APB	ORR, DCR
Zhu 2014	>60	III–IV	38 vs. 38	59.6 vs. 59.8^▼^	CIK+CT	14 days	n.r.	n.r., 4 cycles	ivgtt.	APB	ORR, DCR
Xu 2010	n.r.	III–IV	28 vs. 28	45.0 vs. 42.0^▲^	CIK+CT	12–14 days	IFN-γ, IL-1α, IL-2, OKT-3	(1.0–10.0) × 10^9^, 4 cycles	ivgtt.	APB	ORR, DCR
Xi 2015	≥60	II–IIIb	26 vs. 26	60.0 vs. 62.0^▲^	DC-CIK+CT	7 days	IFN-γ, IL-1, IL-2, OKT-3 (CIK); GM-CSF, IL-4, TNF-α, IL-1 (DC)	(6.0–8.0) × 10^9^, 2 cycles	ivgtt.	APB	QLIR
Yang 2015	n.r.	n.r.	100 vs. 100	70.2 vs. 72.3^▼^	DC-CIK + CT	14 days	n.r.	n.r., 2 cycles	ivgtt.	APB	OS, ORR, DCR, QLIR
Yang 2016	n.r.	n.r.	35 vs. 35	64.9 vs. 65.3^▼^	DC-CIK+CT	14 days	IFN-γ, IL-1, IL-2, OKT-3 (CIK); GM-CSF, IL-4, TNF-α, IL-1 (DC)	5.0 × 10^9^, 2 cycles	ivgtt.	APB	OS, ORR, QLIR
Zhang 2016	≥60	IV	32 vs. 28	56.9 vs. 56.0^▼^	DC-CIK+CT	7 days	n.r.	≥6.0 × 10^9^, 2 cycles	ivgtt.	APB	ORR, DCR, QLIR
Zhang 2017	≥60	II–IIIb	30 vs. 30	64.0 vs. 64.2^▼^	DC-CIK+CT	7 days	IFN-γ, IL-1, IL-2, OKT-3 (CIK); GM-CSF, IL-4, TNF-α, IL-1 (DC)	(6.0–8.0) × 10^9^, 2 cycles	ivgtt.	APB	ORR, DCR, QLIR
Zhao 2015	>70	IIIb–IV	50 vs. 50	55.9 vs. 56.5^▼^	DC-CIK+CT	7 days	n.r.	n.r., 4 cycles	ivgtt.	APB	ORR, DCR, GIAR, LP

Abbreviations: KPS, Karnofsky performance score; n. r., not reported; CT, chemotherapy; Exp, experimental; CIK, cytokine-induced killer cell; DC, dendritic cell; ivgtt., intravenously guttae; APB, autolougs peripheral blood; OS, overall survival; ORR, objective response rate; DCR, disease control rate; QLIR, quality-of-life improved rate; GIAR, gastrointestinal adverse reaction; LP, leukopenia. Regular (▲) and inverted (▼) triangle median and mean age, respectively.

### Methodological quality

**FIGURE 2:**
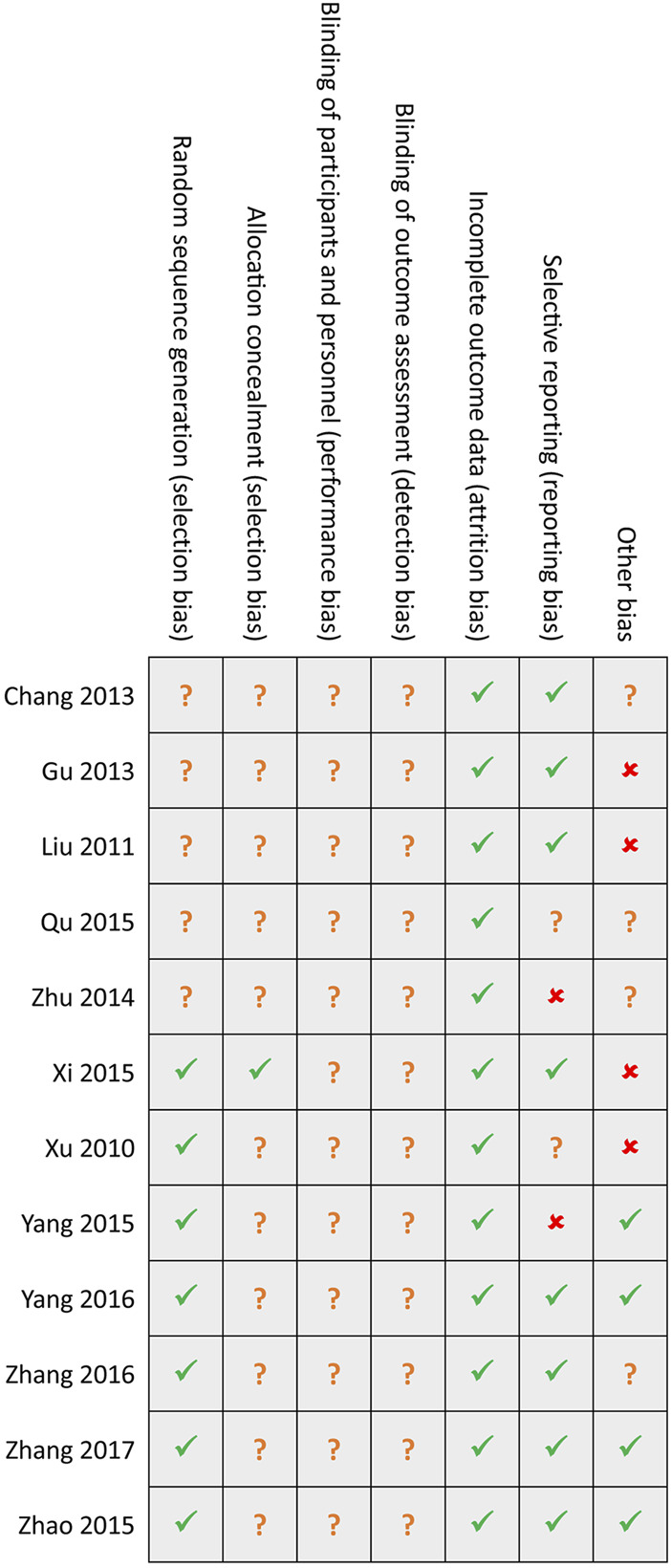
Risk of bias summary. Green hook, yellow question mark, and red cross indicates low, unclear, and high risk, respectively.

Among included 12 studies, seven studies [39–45] generated random sequences using an appropriate method, such as a random number table, but only one study [39] reported the details of concealed random sequences. Not eligible studies reported the details of avoiding performance and detection biases. Attrition bias was assessed as the low risk among all eligible studies. Two studies [42, 46] did not report all anticipated outcomes, and four studies [36, 37, 39, 40] were assessed as high risk due to insufficient sample size. Generally, the overall methodological quality of all included studies was moderate level. Details of the risk of bias in each study are summarized in Figure 2.

### Meta-analysis of OS

Three eligible studies [37, 41, 42] reported OS when comparing immunotherapy plus CT with CT alone, and a meta-analysis suggested that immunotherapy plus CT improved OS among patients with EC (OR 4.10, 95% CI 1.23–13.69, *p* = 0.02, Supplementary Figure S1). Subgroup analysis revealed that CIK+CT (1 RCT, OR 27.00, 95% CI 4.57–159.66, *p* < 0.001) or DC-CIK+CT (2 RCTs, OR 2.10, 95% CI 1.26–3.48, *p* = 0.004) was also better than CT alone for OS (Supplementary Figure S1).

**FIGURE 3:**
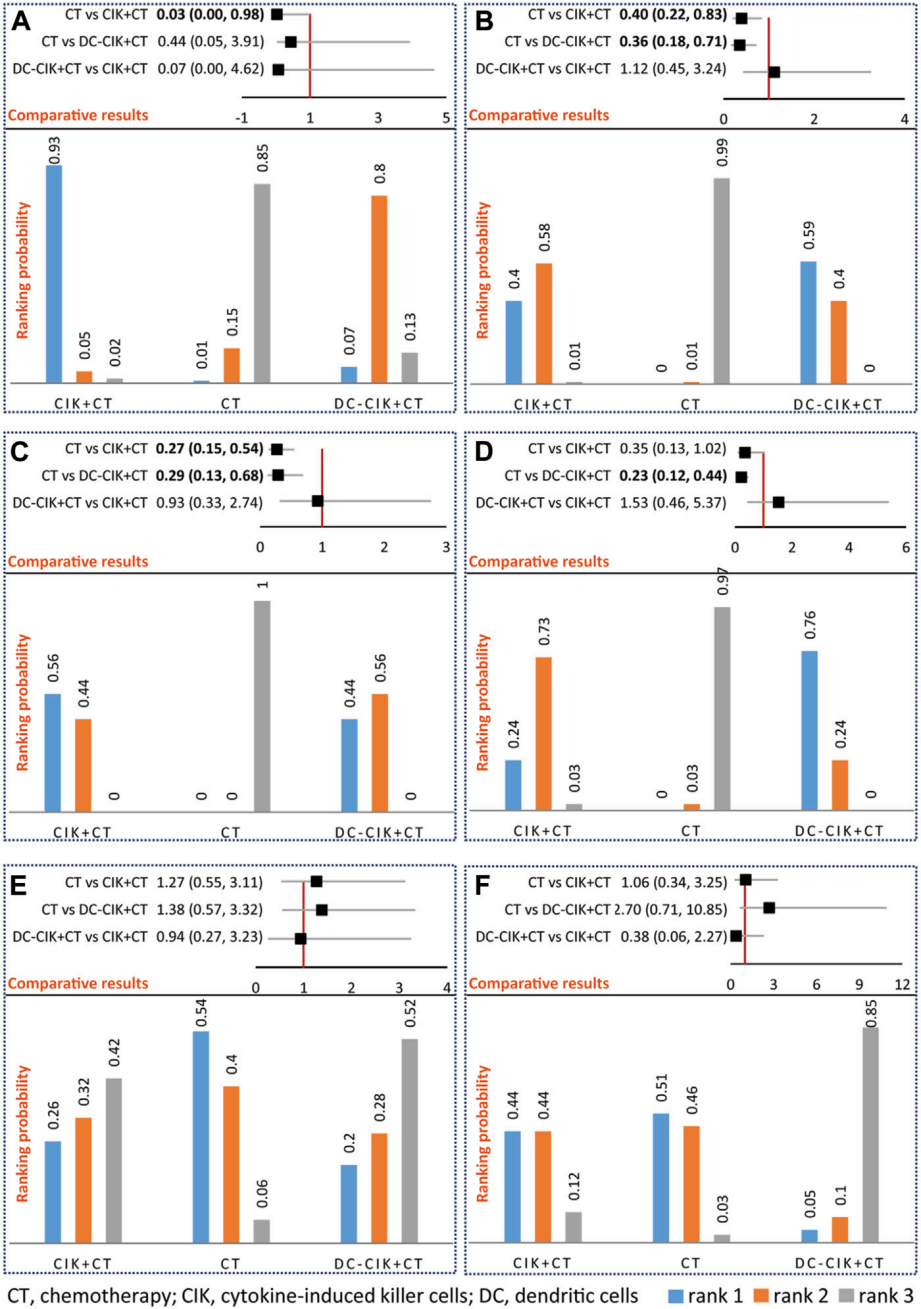
Network meta-analysis of OS **(A)**, ORR **(B)**, DCR **(C)**, QLIR **(D)**, GIAR **(E)**, and leukopenia **(F)**. The upper section represents pooled result of network meta-analysis and the lower section represents ranking probability in an individual figure. For positive outcomes including OS, ORR, DCR, and QLIR, rank 1 indicates the optimal option, rank 2 indicates relatively better option, and rank 3 indicates the worst option. For negative outcomes, including GIAR and leukopenia, rank 1 indicates the worst option, rank 2 indicates a worse option, and rank 3 indicates the optimal option.

Network meta-analysis supported that CIK+CT was superior to CT alone (OR 0.03, 95% CrI 0.00–0.98); however, no statistical difference was detected between DC-CIK+CT and CIK+CT (OR 0.07, 95% CrI 0.00–4.62) or CT alone (OR 0.05, 95% CrI 0.05–3.91) for OS (Figure 3A). Ranking results suggested that CIK+CT was the optimal option, followed by DC-CIK+CT and CT alone (Figure 3A).

### Meta-analysis of ORR

A total of 11 studies [35–38, 40–46] reported ORR, and meta-analysis suggested that immunotherapy plus CT (OR 2.72, 95% CI 1.79–4.11, *p* < 0.001) was better than CT alone for ORR. Subgroup analysis revealed that CIK+CT (6 RCTs, OR 2.79, 95% CI 1.47–5.30, *p* = 0.002) or DC-CIK+CT (5 RCTs, OR 2.60, 95% CI 1.45–4.67, *p* = 0.001) was also better than CT alone for OS (Supplementary Figure S2).

Network meta-analysis supported that CIK+CT (OR 0.40, 95% CrI 0.22–0.83) or DC-CIK+CT (OR 0.36, 95% CrI 0.18–0.71) was superior to CT alone; however, no statistical difference was detected between DC-CIK+CT and CIK+CT (OR 1.12, 95% CrI 0.45–3.24) (Figure 3B). Ranking results suggested that DC-CIK+CT was the optimal option, followed by CIK+CT and CT alone (Figure 3B).

### Meta-analysis of DCR

The meta-analysis of 10 studies [35–38, 40, 42–46] suggested that immunotherapy plus CT (OR 3.45, 95% CI 2.32–5.14, *p* < 0.001) was better than CT alone for DCR (Supplementary Figure S3). Subgroup analysis revealed that CIK+CT (6 RCTs, OR 3.59, 95% CI 2.16–5.98, *p* < 0.001) or DC-CIK+CT (4 RCTs, OR 3.24, 95% CI 1.71–6.15, *p* < 0.001) was also better than CT alone (Supplementary Figure S3).

Network meta-analysis supported that CIK+CT (OR 0.27, 95% CrI 0.15–0.54) or DC-CIK+CT (OR 0.29, 95% CrI 0.13–0.68) was superior to CT alone for DCR; however, no statistical difference was detected between DC-CIK+CT and CIK+CT (OR 0.93, 95% CrI 0.33–2.74) (Figure 3C). Ranking results suggested that CIK+CT was the optimal option, followed by DC-CIK+CT and CT alone (Figure 3C).

### Meta-analysis of QLIR

Meta-analysis of 8 studies [35–37, 39, 41–44] suggested a better QLIR among patients treated by immunotherapy plus CT (OR 3.54, 95% CI 2.31–5.41, *p* < 0.001) compared with CT alone (Supplementary Figure S4). Subgroup analysis revealed that CIK+CT (3 RCTs, OR 2.42, 95% CI 1.09–5.40, *p* = 0.03) or DC-CIK+CT (5 RCTs, OR 4.10, 95% CI 2.48–6.76, *p* < 0.001) was also better than CT alone (Supplementary Figure S4).

Network meta-analysis supported that DC-CIK+CT (OR 0.23, 95% CrI 0.12–0.44) was superior to CT alone for QLIR; however, no statistical difference was detected between CIK+CT and DC-CIK+CT (OR 1.53, 95% CrI 0.46–5.37) or CT alone (OR 0.35, 95% CrI 0.13–1.02) (Figure 3D). Ranking results suggested that DC-CIK+CT was the optimal option, followed by CIK+CT and CT alone (Figure 3D).

### Meta-analysis of AEs

Among 12 included studies, only 3 studies [35, 37, 45] reported AEs, including GIAR and QLIR. The meta-analysis did not detect a significant difference in any comparisons (Supplementary Figure S5), supported by network meta-analysis (Figures 3E, F). Meanwhile, network meta-analysis did not detect a statistical difference between CIK+CT and DC-CIK+CT for any AEs (Figures 3E, F). Ranking results suggested that DC-CIK+CT was the optimal option, followed by CIK+CT and CT alone regarding AEs (Figures 3E, F).

## Discussion

EC remains one of the most common digestive malignant tumors worldwide [1]. Although surgery, radiotherapy, and chemotherapy are most widely used for EC, their application is limited by failing to thoroughly eliminate tumor cells, drug resistance, and other adverse effects [4, 5]. Immunotherapy has rapidly developed [3] since immunodeficiency is often considered a decisive factor in the recurrence and metastasis of EC patients [6]. Among numerous immunotherapy regimes, CIK and the combination of DC and CIK were more frequently applied in clinical practice due to advantages such as easy access [5] and cytotoxicity is neutral on MHC [14]. Multiple RCTs and meta-analyses have established the treatment efficacy and safety of immunotherapy built on CIK or DC-CIK plus CT for the treatment of patients with EC; however, it’s unclear whether the presence of a difference between CIK and DC-CIK because of a trial directly comparing these two regimes is unlikely to occur. In this network meta-analysis, we further established the role of immunotherapy based on CIK or DC-CIK in treating EC patients, although no difference is detected in terms of AEs. We also establish the beneficial therapeutic value of CIK or DC-CIK for the treatment of EC patients compared with CT alone. It’s noted that CIK and DC-CIK are comparable in treatment efficacy and safety among patients with EC.

Currently, three meta-analyses [18–20] have been identified to compare immunotherapy built on CIK or DC-CIK with CT alone for treating patients with EC. Liu et al. conducted a meta-analysis of 11 RCTs to investigate the role of CIK/DCs-CIK immunotherapy in treating Chinese EC patients and suggested that the combination of CIK/DC-CIK immunotherapy and CT is safe and markedly prolongs survival time, enhances immune function, and improves the treatment efficacy for EC [19]. In 2021, Ling et al. conducted a meta-analysis of 13 RCTs to determine the efficacy and safety of CIK of adoptive immunotherapy combined with CT for the treatment of EC. They indicated that CIK cells of adoptive immunotherapy combined with CT could improve the clinical efficacy of EC patients, improve their quality of life and enhance their immune response [18]. In the same year, another meta-analysis of 17 RCTS revealed that the combination therapy of CIK/DC-CIK immunotherapy and CT enhances the immune function and improves the therapeutic efficacy of patients with EC [20]. In this network meta-analysis, we only focused on clinical outcomes, including treatment efficacy and AEs, and conventional pairwise meta-analysis obtained consistent results with previous meta-analyses. The comparison of the present network meta-analysis and previous meta-analyses is summarized in Table 2. It’s noted that we only considered studies that compared CIK/DC-CIK plus CT with CT alone to be eligible, and thus heterogeneity resulting from regimes could be reduced. As a result, more reliable and robust results are generated from our study.

**TABLE 2:** Comparison of the present network meta-analysis and previous meta-analyses.

Study	IT+CT vs. CT alone						CIK+CT vs. CT alone						DC-CIK+CT vs. CT alone						CIK+CT vs. DC-CIK+CT					
	OS	ORR	DCR	QLIR	GIAR	LP	OS	ORR	DCR	QLIR	GIAR	LP	OS	ORR	DCR	QLIR	GIAR	LP	OS	ORR	DCR	QLIR	GIAR	LP
Liu 2017	✓	✓	✓	✓	?	?	n.a.						n.a.						n.a.					
Ling 2021	✓	✓	✓	✓	?	?	n.a.						n.a.						n.a.					
Yuan 2021	✓	✓	✓	✓	?	?	n.a.						n.a.						n.a.					
The present study	✓	✓	✓	✓	?	?	✓	✓	✓	?	?	?	?	✓	✓	✓	?	?	?	?	?	?	?	?

Notes: n.a., not available. “✓” indicates benefiting to the combination regime of immunotherapy and CT, and “?” indicates no difference between two regimes.

Additionally, we investigated the potential treatment value of CIK or DC-CIK for treating patients with EC based on subgroup analysis, which does not occur in previous meta-analyses. In the pairwise meta-analysis, the treatment efficacy of CIK or DC-CIK in treating EC is suggested. However, a significant difference between DC-CIK+CT and CT alone for OS and a significant difference between CIK+CT and CT alone for ORR is not retained in network meta-analysis, which may result from the limited number of included studies for individual comparison. More importantly, to date, no study has been conducted to directly compare CIK with DC-CIK for the treatment of EC patients, and thus it’s unclear whether there is a difference between these two regimes. This network meta-analysis first investigates the comparative efficacy and safety between CIK+CT and DC-CIK+CT in EC patients. Interestingly, our network meta-analysis does not detect the difference between CIK+CT and DC-CIK+CT for all outcomes among EC patients.

Although this network meta-analysis generates more reliable and robust findings for decision-making, several limitations must be further interpreted. First, 12 eligible studies with a limited sample size were included in the final analysis, which greatly impaired the reliability of pooled results. Second, we did not conduct a publication bias test and small study effects due to a limited number of eligible studies for individual comparison. And thus, we must consider the possible impact of publication bias on the pooled results when interpreting pooled results. Third, a closed-loop cannot be available for any outcomes, and thus we could not evaluate inconsistency between direct and indirect comparisons with star-shaped network geometry. Forth, we cannot design subgroup analysis to investigate treatment efficacy and safety due to the limited number of eligible studies, although variations are detected in cell dose, KPS, and tumor stage. Fifth, all eligible studies are conducted in China; thus, these findings should be cautiously implemented in other cultural settings. Sixth, we also must acknowledge that variations in the sources of CIK and DC cells were not further investigated due to insufficient eligible studies, which might negatively affect the reliability of the pooled results.

## Conclusion

Our network meta-analysis further demonstrated that immunotherapy plus CT is better than CT alone for treating EC patients. Meanwhile, CIK+CT or DC-CIK+CT is superior to CT alone for OS and treatment efficacy. However, CIK+CT and DC-CIK+CT may be statistically comparable for EC patients. Therefore, we concluded that CIK cell treatment is superior to CT alone in treating patients with EC. However, our findings of comparing CIK-CT and DC-CIK+CT are only obtained from indirect evidence, and therefore, it is undoubtedly necessary to conduct studies to directly compare CIK-CT with DC-CIK+CT in EC patients in the future.

## Data Availability

The original contributions presented in the study are included in the article/Supplementary Material, further inquiries can be directed to the corresponding author.
